# Analysis of exosome-derived microRNAs reveals insights of intercellular communication during invasion of breast, prostate and glioblastoma cancer cells

**DOI:** 10.1080/19336918.2021.1935407

**Published:** 2021-06-22

**Authors:** Francesca Lessi, Paolo Aretini, Milena Rizzo, Mariangela Morelli, Michele Menicagli, Sara Franceschi, Chiara Maria Mazzanti

**Affiliations:** a Fondazione Pisana per La Scienza, Pisa, Italy; b Institute of Clinical Physiology (IFC), CNR, Pisa, Italy

**Keywords:** Exosomes, miRNAs, tumor invasion, breast cancer, glioblastoma, prostate cancer

## Abstract

MiRNAs represent a mechanism that regulates gene expression in many pathological conditions. Exosomes are known to be secreted from all types of cells, and the exosomes-released molecules are crucial messengers that can regulate cellular processes. We investigated the miRNAs content of exosomes released by cancer cells during the invasion . An invasion stimulus has been generated through scratches created on the confluent cells of cancer cell lines: glioblastoma, breast and prostate cancers.

Several miRNAs were found to be significantly differentially abundant during the cell invasion , both in common among different cell lines and exclusive. Understanding the language codes among cells involved in invasion can lead to the development of therapies that can inhibit cellular communication, slowing or eventually stopping their activity.

## Introduction

One of the most overwhelming principles in the development of a tumor is certainly the ability to invade surrounding tissues as also indicated by the Hallmarks of Cancer [1]. The study of cell invasion in cancer research is of particular interest as the main cause of death in cancer patients is related to the tumor capability to invade and metastasize [2]. At the base of the invasion process, the connection between the cells becomes essential in order to understand why some cells begin to spread to the adjacent tissues. Communication between cells becomes crucial in this situation. There are several approaches by which cells can communicate with each other, such as by a direct interaction through membrane receptors or ligands, or by releasing soluble molecules (growth factors, cytokines and chemokines). In the last few years, another way of connecting cells has been identified, that is, through the content of extracellular vesicles (EVs) such as exosomes.

Exosomes are small endosome-derived vesicles ranging in size from 40–150 nm in diameter that are distributed in various biological fluids [3]. These vesicles can contain nucleic acids, proteins and lipids. Exosomes are involved in multiple biological functions, including regulation of immune responses, antigen presentation, intercellular communication and tumor proliferation [4]. In the oncology field, exosomes have been studied for many years, since the complex exosome-mediated crosstalk between cancer and non-cancer cells seems to be involved in every step of tumoral progression, from tumor growth to cell dissemination [5].

Tumor exosomes can contain functional mRNAs, small RNAs as microRNAs (miRNAs), and proteins that may be involved in promoting invasion [6]. In cell–cell communication it is interesting to note that EVs (such as exosomes) derived from cancer cells are capable of transferring miRNA, mRNA and proteins not only to adjacent cells of the same type but also to distant cells [7].

MiRNAs are short endogenous non coding-RNAs (21–23 nucleotides) that bind to the untranslated regions of target genes causing translational repression of the target gene or stimulating rapid degradation of the target transcript [8]. MiRNAs are able to regulate signaling pathways, involved in cell development and differentiation, cell proliferation, and apoptosis [9].

Recently, miRNAs encapsulated in secreted EVs have been identified in the extracellular space. Mature miRNAs involved in intercellular communication are released from most cells, often within EVs, and disseminate through the extracellular fluid to reach remote target cells, including tumor cells, where they can act as tumor suppressors or oncogenes, depending on the targets that they regulate [10].

In this study, we decided to focus our attention on the communication between cells subjected to stimuli that promote invasion. As not described before, through next-generation sequencing methods, we evaluated the overall expression profile of miRNAs derived from exosomes in different types of cancer cells (breast, prostate and glioblastoma tumor) subjected to the same stimuli of invasion. We chose these cancer cell lines (MDA-MB-231, PC3 and T98G) for their aggressive and potentially metastatic behavior, focusing on three different tumors just to understand if the invasion process had or had not a common basis in cellular communication. Our aim was to investigate similarities among miRNAs signatures contained into the exosomes released by cancer cell lines, exposed to a stimulus of invasion. The possibility of similar miRNA signatures in cancers of different types could pinpoint at biomarkers of tumor invasion as a universal concept. At the same time, each tumor type could carry a cell-specific exosome-derived miRNA signature, resulting in a unique concept of invasion mechanism for different types of cancers.

## Materials and methods

### Cell cultures

The human cancer cell lines used in this experiment are breast cancer cells MDA-MB-231 (ATCC, LGC Standards, Manassas, VA, USA), glioblastoma cells T98G (ATCC, LGC Standards, Manassas, VA, USA) and prostate cancer cells PC3 (ATCC, LGC Standards, Manassas, VA, USA). MDA-MB-231 were cultured in high glucose DMEM medium (Thermo Fisher Scientific, Waltham, MA, USA), T98G in low glucose DMEM medium and PC3 in HAM’s F12K medium (Thermo Fisher Scientific, Waltham, MA, USA). All the media were supplemented with 10% FBS (Thermo Fisher Scientific, Waltham, MA, USA), 100 U/ml penicillin (Thermo Fisher Scientific, Waltham, MA, USA) and 100 μg/ml streptomycin (Thermo Fisher Scientific, Waltham, MA, USA). All the cell cultures were incubated at 37°C in an atmosphere of 5% CO_2_.

### Scratches creation for invasive stimulus

**Figure 1.**
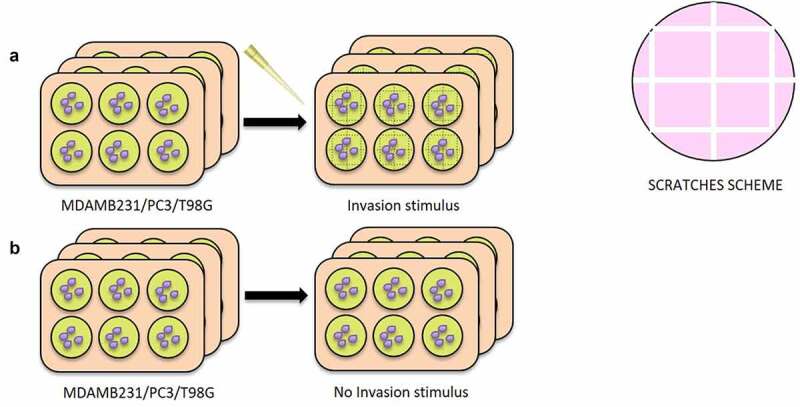
**Creation of the scratches for Invasive Stimulus (IS) experiment**. 6 plates were used for all cell lines (MDAMB231, T98G and PC3), 3 with the IS and 3 without IS. In A) it is shown the set-up of the experiment: when the cells reached confluence, 6 intersected scratches were perpendicularly made with a tip, 3 vertically and 3 horizontally, in the 3 IS plates. In B) 3 plates used as negative control, without the creation of the scratches

Five hundred thousand cells were plated on 6-well plates for all cancer cell lines. Three plates were used to carry out the invasion experiment and three plates as control, in triplicate for each cell line. When cells reached confluence, six intersected scratches were perpendicularly made with a tip, three vertically and three horizontally as shown in Figure 1 to simulate a situation of invasion, mimicking a typical wound healing assay [11]. Starting with the number of cells at confluence and the area of one well of a 6-well plate, and subtracting the number of cells that are inside the scratches, the number of cells that have the potential to invade per well is approximately 1 × 10^6^. After about 8 hours, the medium was changed with complete medium lacking FBS to avoid the contamination of exosomes normally contained in FBS.

### Wound healing assay with conditioned medium containing exosomes

Medium containing exosomes, released from cancer cells stimulated to invade (INV-ex), was used to carry out a wound healing assay experiment to investigate their potential role as inducer of invasion. A conditioned medium with exosomes released from confluent cancer cells (CTRL-ex) was used as a negative control.

**Figure 2.**
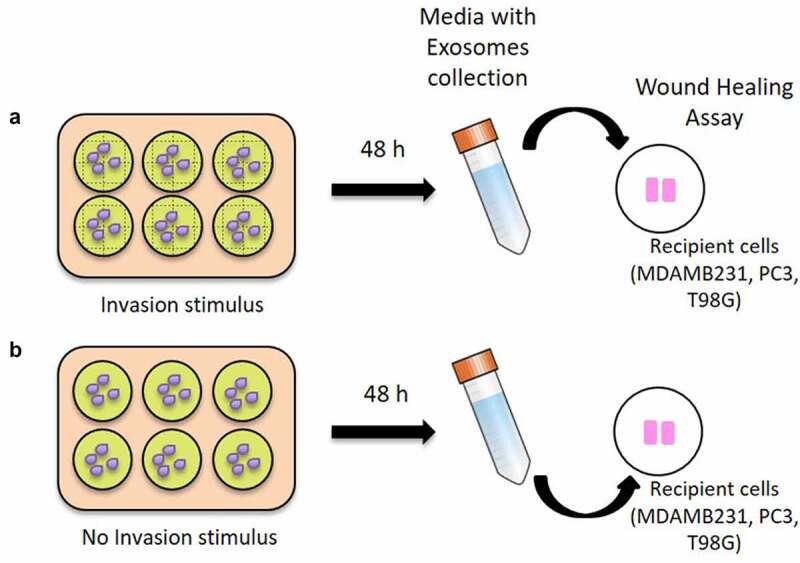
**Wound healing assay with conditioned medium containing exosomes**. In A) it is shown the harvesting of the conditioned medium with exosomes released 48 hours after the scratches creation (INV-ex) for each cancer cell line and the transfer onto the respective cells (MDA-MB-231, PC3 and T98G) seeded in the IBIDI dishes. In B) a conditioned medium with exosomes released from confluent cancer cells (CTRL-ex) was used, as a negative control. Wound healing assay was performed for all cancer cell lines using IBIDI dishes

In particular, 30.000 cells of MDAMB231, T98G and PC3 were seeded in each insert of a dish with a culture-insert 2 well (IBIDI, Martinsried, Germany). Conditioned medium therefore was transferred onto the cells in the IBIDI dish, immediately after the insert removal. After 24 hours of incubation, the cells, which migrated into the wounded area or protruded from the border of the wound were visualized and photographed under an inverted microscope. The whole experiment is outlined in Figure 2. All the images were collected and analyzed with Image J Tool. The graphs were analyzed with GraphPad Prism 9.1, and the p-value was calculated through unpaired T test (two-tailed).

### Exosomes isolation

After 48 hours from scratching, exosomes were isolated from the cell culture medium using the Total Exosomes Isolation (from cell culture medium) Kit (Thermo Fisher Scientific, Waltham, MA, USA) starting from 10 ml of medium per sample and resuspended in 200 μl of PBS 1X. Exosomes were also isolated from culture medium of confluent cells not subjected to scratches. The presence of exosomes was verified using anti-CD63 antibody (Thermo Fisher Scientific, Waltham, MA, USA) with a Western Blot detection on all the three cancer cell lines (data not shown).

### Small RNA isolation

Small RNAs were isolated from exosomes using Total Exosome RNA and Protein Isolation Kit (Thermo Fisher Scientific, Waltham, MA, USA). All small RNA samples obtained were then quantified with QubitTM 4 fluorometer using Qubit miRNA assay kit (Thermo Fisher Scientific, Waltham, MA, USA). Moreover, the quality of samples was verified with TapeStation System (Agilent, Headquarters, Santa Clara, CA, USA).

### MiRNome libraries

MiRNome libraries were prepared for all samples using TruSeq Small RNA Library Preparation kit (Illumina, San Diego, CA, USA) and run on a MiSeq Instrument (Illumina, San Diego, CA, USA).

### MiRNome data analysis

After performing the Quality Control (QC) of raw data as the initial step of routine miRNA-seq workflow, we carried out miRNAs analysis using miARmaSeq (1.7.3 version, http://miarmaseq.idoproteins.com/). To analyze the data, we set the configuration file using Bowtie1 as aligner feature. Differential expression of reads count was evaluated by using EdgeR (3.1 version). Significance statistical was quantified using the False Discovery Rate (FDR) which is estimated by EdgeR using the Benjamini–Hochberg method. FDR values less than 0.05 were considered statistically significant. The full miARma-Seq analysis flow is available as supplementary material (Supplementary file 1). Heatmap was generated using pheatmap R package. The pathways analysis on differentially abundant miRNAs was conducted using webtool mirPath v.3 (http://snf-515788.vm.okeanos.grnet.gr/).

### miRNA targets prediction and pathway analysis

Target genes for differentially abundant exosome-derived miRNAs were identified using the target prediction tool DIANA-microT web server V5.0 11, in particular MirPath v3 tool. This is a miRNA pathway analysis web-server that utilize predicted miRNA targets provided by the DIANA-microT-CDS algorithm or experimentally validated miRNA interactions derived from DIANA-TarBase. These interactions can be subsequently combined with sophisticated merging and meta-analysis algorithms. The first top 10 differentially abundant miRNAs for each cancer cell line were included in the software, setting the threshold for miTG (miRNA targeted genes) score to 0.9 and doing the research among the genes related to KEGG (Kyoto Encyclopedia of Genes and Genomes) pathways with description that matches ‘cancer invasion’. The miTG score is a general score for the predicted interaction, the closer to 1, the greater the confidence. The heatmaps were obtained using the R library, Pheatmap.

### Gene validation by website dataset analysis

GEPIA web-tool (Gene Expression Profiling Interactive Analysis) (http://gepia.cancer-pku.cn/) based on TCGA and GTEx data retrieved from the UCSC Xena server [12] was used to validate miRNA expression results. We used GEPIA tool to investigate the data obtained after DIANA-TarBase analysis on the genes targeted by multiple differentially abundant miRNAs in our cancer cell lines submitted to IS. GEPIA dataset included: 1085 breast cancers and 291 normal breast tissues; 492 prostate cancers versus 152 normal prostate tissues; 163 glioblastoma samples and 207 normal brain tissues. Glioblastoma data was also investigated by using Ivy Glioblastoma Atlas Project (https://glioblastoma.alleninstitute.org/static/home) [13] which provides in situ hybridization (ISH) and RNA sequencing (RNA-Seq) data, correlating gene expression across anatomical structures in glioblastoma. The Ivy database consists in 42 Infiltrating Tumor sampled by reference histology, 111 Cellular Tumor portions sampled by low expression of gene TNFAIP3, 66 Pseudopalisading Cells around Necrosis sampled by high expression of gene TNFAIP3, 22 Hyperplastic Blood Vessels portions in cellular tumor sampled by high expression of gene TGFBR2 and 28 Microvascular Proliferation sampled by high expression of gene TGFBR2.

## Results

### Effect of INV-ex conditioned medium in cell invasion

**Figure 3.**
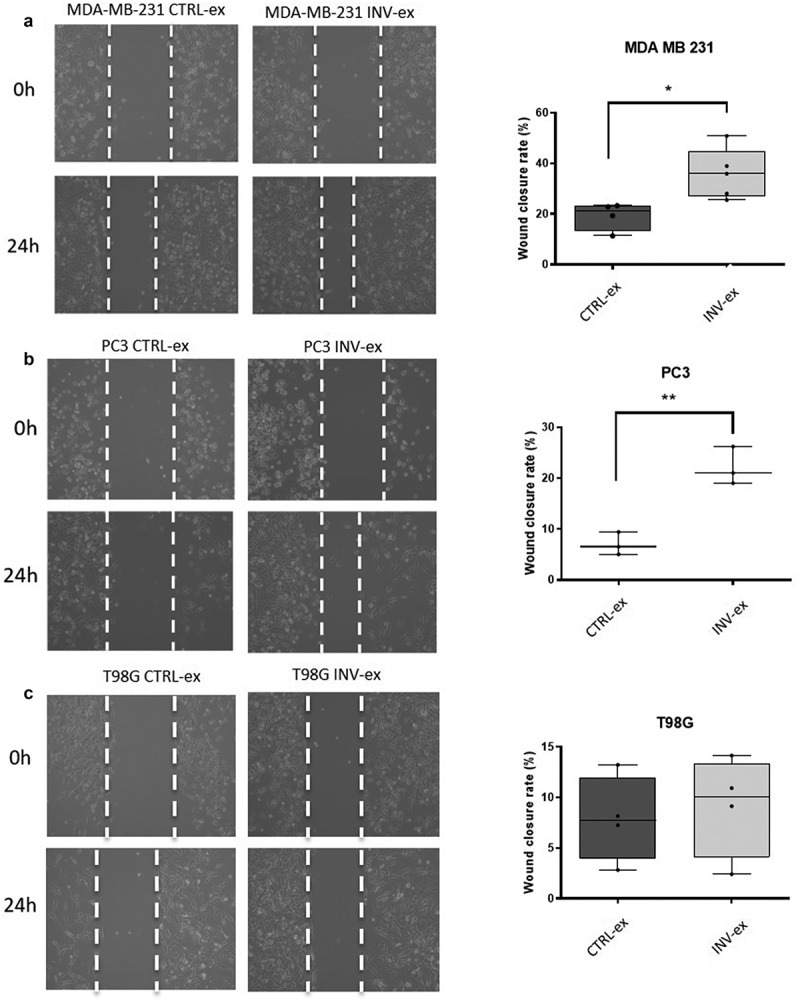
**Wound healing assay**. In A) it is shown the image of the wound at 0 h and 24 h after the exposure to conditioned INV-ex and CTRL-ex medium in MDA-MB-231. An increase of invasion is seen in MDA-MB-231 exposed to the INV-ex medium. On the right-hand side the histogram shows the percentage of wound closure calculated using Image J Tool of different fields (p = 0.0211); in B) it is shown the wound closure at 0 h and after 24 h in PC3 exposed to INV-ex and CTRL-ex medium. The histogram reports the increased percentage of wound closure in the PC3 exposed to INV-ex medium (p = 0,0038); in C) it is reported the wound closure at 0 h and after 24 h for T98G cells treated with conditioned INV-ex and CTRL-ex medium. A decrease in cell invasion in T98G exposed to CTRL-ex medium is detectable but without a statistical significance (p = 0.7)

The cells that received the INV-ex medium showed a greater invasion capacity, meant as a higher percentage of wound closure for all the cancer cell lines compared to the ones that received the CTRL-ex medium as shown in Figure 3. The increase in wound closure resulted statistically significant for MDA-MB-231 breast and PC3 prostate cancer cell lines with p = 0.0211 and p = 0.0038, respectively, when exposed to the INV-ex medium compared to the CTRL-ex medium. In the same way, T98G glioblastoma cell lines showed an increase in wound closure after exposure to conditioned INV-ex medium but did not reach statistical significance (p = 0.7).

### Exosome-derived differentially abundant miRNAs

**Figure 4.**
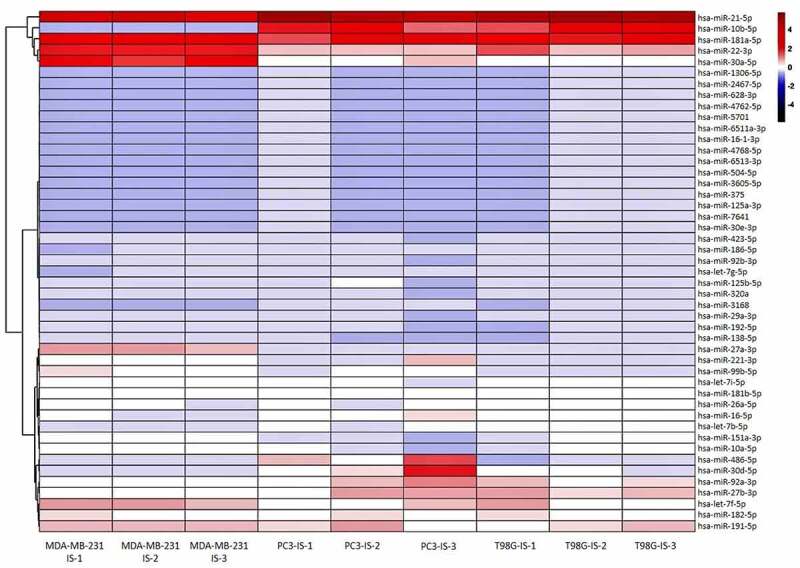
**Exosomes-derived differentially abundant miRNAs**. Heatmap generated with R showing the statistical significant differentially abundant exosomes-derived miRNAs in all three replicates (1, 2 and 3) of the cell lines subjected to the IS

Exosomes were isolated and the internal miRNOMA profile was investigated to identify the presence of specific miRNAs accountable for the invasion process and common to the different culture cancer cell lines subjected to the invasion stimuli (IS) compared to the confluent cancer cell that did not receive any stimulus (noIS). Table 1S in the supplementary materials, summarizes the quality of the sequencing and reads obtained. All data obtained are available in GEO/SRA (http://www.ncbi.nlm.nih.gov/bioproject/720327). A heatmap, based on all miRNAs differentially abundant in a statistically significant way, was generated to more easily visualize the trend of miRNAs in the different cell lines tested, as shown in Figure 4.

**Table 1.** Differentially abundant (p < 0.05) exosome-derived miRNAs in MDA-MB-231 breast cancer cell line after IS

miRNA	Length	PValue	FDR	logFC
hsa-miR-181a-5p	46	2.19E-16	4.90E-14	5.55
hsa-miR-486-5p	44	3.53E-15	3.96E-13	−4.1
hsa-miR-27a-3p	21	1.28E-13	9.53E-12	1.29
hsa-miR-22-3p	22	4.77E-12	2.67E-10	3.8
hsa-miR-182-5p	24	3.56E-10	1.60E-08	1.21
hsa-miR-92a-3p	44	1.51E-08	5.62E-07	1.17
hsa-let-7 f-5p	44	3.99E-08	1.28E-06	3.67
hsa-miR-30a-5p	22	8.30E-08	2.32E-06	4.18
hsa-miR-21-5p	22	2.46E-07	6.13E-06	2.45
hsa-let-7i-5p	22	4.73E-07	1.06E-05	1.13
hsa-miR-191-5p	23	5.60E-07	1.14E-05	4.46
hsa-miR-27b-3p	21	7.82E-07	1.46E-05	1.12
hsa-miR-26a-5p	44	3.88E-06	6.69E-05	1.09
hsa-miR-151a-3p	21	5.42E-06	8.68E-05	0.1
hsa-miR-16-5p	44	1.39E-05	0.0002	0.1
hsa-miR-221-3p	23	0.0002	0.003	2.55
hsa-miR-181b-5p	46	0.0003	0.004	3.47
hsa-miR-192-5p	21	0.004	0.06	9.45
hsa-miR-138-5p	46	0.005	0.068	9.43
hsa-miR-423-5p	23	0.01	0.115	9.31
hsa-miR-92b-3p	22	0.016	0.179	9.03
hsa-miR-30d-5p	22	0.018	0.179	0.22
hsa-miR-10a-5p	23	0.018	0.17	1.99
hsa-miR-99b-5p	22	0.023	0.218	1.9
hsa-miR-29a-3p	22	0.029	0.252	0.87
hsa-miR-125b-5p	44	0.029	0.252	8.65
hsa-let-7 g-5p	22	0.037	0.314	8.55
hsa-miR-320a	22	0.042	0.337	8.61
hsa-miR-186-5p	22	0.043	0.337	8.57

**Figure 5.**
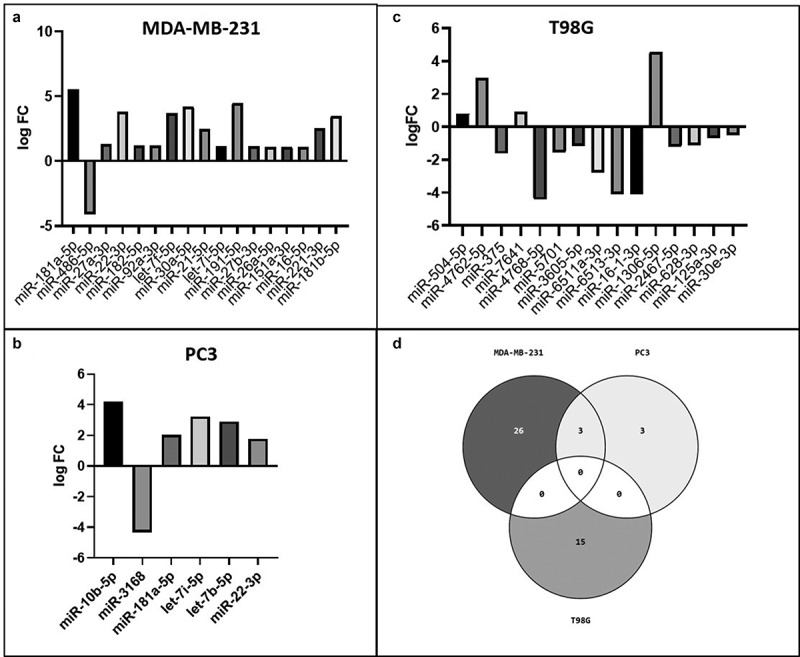
**Exosome-derived differentially abundant miRNAs**. In A) the differentially abundant exosome-derived miRNAs with FDR<0.05 in MDA-MB-231 are shown. In B) and C) are shown the differentially abundant exosome-derived miRNAs in PC3 and T98G cells, respectively; in D) the Venn diagram shows the miRNAs in common among the 3 cancer cell lines

In MDA-MB-231 breast cancer cells, the level of 29 exosome-derived miRNAs, listed in Table 1, were found to be significantly different between the IS and noIS groups (p < 0.05). In Figure 5a are shown 17 differentially abundant miRNAs with FDR < 0.05: the level of these miRNAs was increased in the IS group, except for miR-486-5p that was decreased.

In PC3 prostate cancer cells, 6 exosome-derived miRNAs, listed in Table 2, were found to be significantly differentially abundant between the IS and noIS groups with p < 0.05. The level of all these 6 miRNAs was increased in the IS group, except for miR-3168 that was decreased (Figure 5b).

**Table 2.** Differentially abundant (p < 0.05) exosome-derived miRNAs in PC3 prostate cancer cell line after IS

miRNA	Length	PValue	FDR	logFC
hsa-miR-10b-5p	23	5.59E-07	4.25E-05	4.19
hsa-miR-3168	17	0.004	0.18	−4.34
hsa-miR-181a-5p	46	0.008	0.21	2.03
hsa-let-7i-5p	22	0.02	0.39	3.23
hsa-let-7b-5p	22	0.03	0.43	2.9
hsa-miR-22-3p	22	0.034	0.43	1.77

The differentially abundant miRNAs isolated from the exosomes derived from T98G glioblastoma cell lines were composed of 15 miRNAs, listed in Table 3, which had statistical significance, p < 0.05. Four of these 15 miRNAs showed higher levels in the IS group (miR-504-5p, miR-4762-5p, miR-7641 and miR-1306-5p), while the others behaved in the opposite way (Figure 5c). A Venn diagram (Figure 5d) was constructed to show the common miRNAs among the 3 different cell lines. T98G did not show miRNAs in common with the other cell lines.

**Table 3.** Differentially abundant (p < 0.05) exosome-derived miRNAs in T98G glioblastoma cell line after IS

miRNA	Length	PValue	FDR	logFC
hsa-miR-504-5p	22	0.005	1	0.82
hsa-miR-4762-5p	21	0.007	1	2.98
hsa-miR-375	22	0.008	1	−1.62
hsa-miR-7641	38	0.017	1	0.91
hsa-miR-4768-5p	22	0.018	1	−4.42
hsa-miR-5701	38	0.023	1	−1.54
hsa-miR-3605-5p	23	0.026	1	−1.17
hsa-miR-6511a-3p	88	0.027	1	−2.79
hsa-miR-6513-3p	21	0.031	1	−4.11
hsa-miR-16-1-3p	22	0.031	1	−4.11
hsa-miR-1306-5p	22	0.031	1	4.57
hsa-miR-2467-5p	23	0.034	1	−1.21
hsa-miR-628-3p	21	0.035	1	−1.1
hsa-miR-125a-3p	22	0.036	1	−0.69
hsa-miR-30e-3p	22	0.036	1	−0.5

### MDA-MB-231 breast and PC3 prostate cancer cells share three differentially abundant miRNAs.

**Figure 6.**
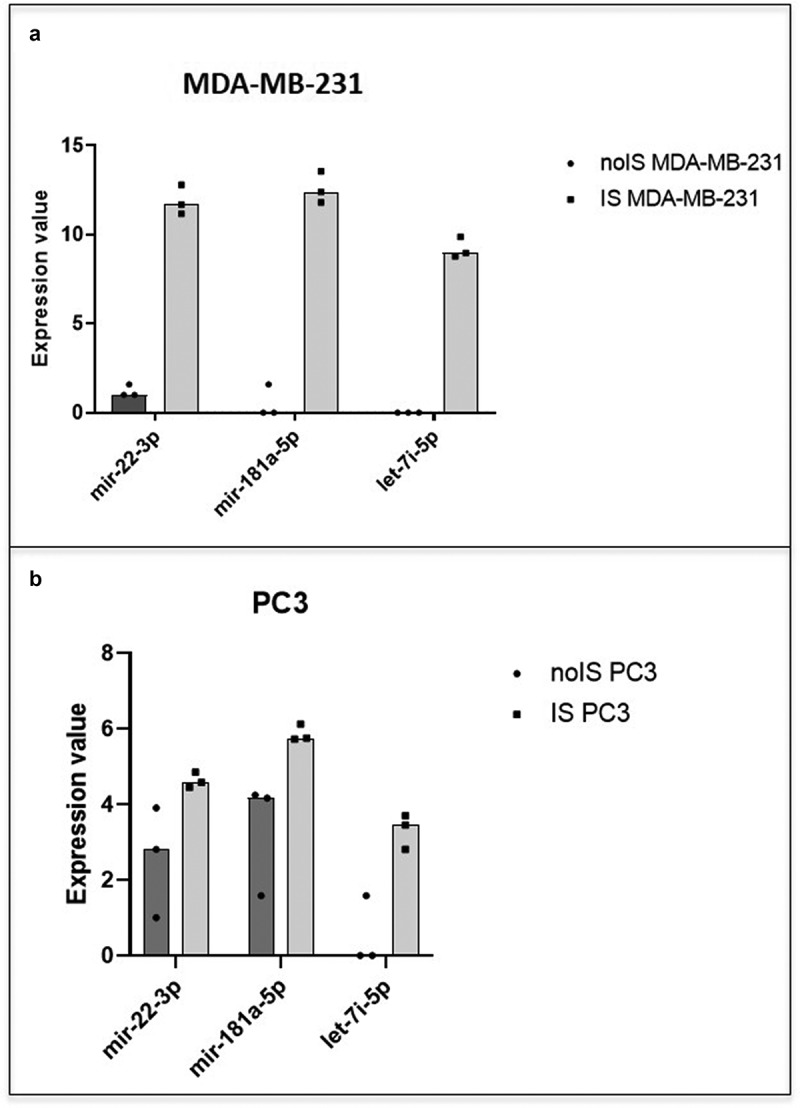
**MDA-MB-231 and PC3 miRNAs content similarity**. The scatter dot plots show the trend of the 3 miRNAs (mir-22-3p, mir-181a-5p and let-7i-5p) in common between MDA-MB-231 (a) and PC3 cells (b). In black the expression value in noIS condition and in gray after IS

As reported in Figure 6a and B, MDA-MB-231 and PC3 cell lines show a statistically significant higher level of the same 3 miRNAs, such as miR-181a-5p, miR-22-3p and let-7i-5p in the IS group. In addition, we found, between the two different cell lines, several miRNAs belonging to the same family such as:

- miR-10b-5p and miR-10a-5p (miR-10 family), higher, respectively, in the IS group of PC3 and MDA-MB-231 cells (Tables 1 and Tables 2).
- Two let-7 family’s miRNAs (let-7i-5p and let-7b-5p) and 3 miRNAs of the same family (let-7 f-5p, let-7i-5p and let-7 g-5p) were higher, respectively, in the IS group of MDA-MB-231 and PC3 (Tables 1 and Tables 2).

### miRNA targets prediction analysis

Using the target prediction tool DIANA-microT web server V5.0 [14], we performed a search for the genes targeted by the differentially abundant exosome-derived miRNAs.

We obtained for MDA-MB-231, PC3 and T98G cell line, 296, 82, and 90 target genes, respectively (Tables 2S, Tables 3S and Tables 4S of supplementary data).

**Table 4.** **Targeted pathways in MDA-MB-231 breast cancer cell line**. List of significantly targeted molecular pathways with genes involved

KEGG Pathway	miRNA	p Value	Genes
Mucin type O-Glycan biosynthesis	hsa-miR-27a-3p	0.0059	GALNT5
	hsa-let-7 f-5p	6.7683E-06	GALNT1
	hsa-miR-30a-5p	1.6772E-10	GALNT7
	hsa-let-7i-5p	8.187E-06	GALNT1
Fatty acid degradation	hsa-miR-486-5p	9.52E-15	EHHADH
Fatty acid metabolism	hsa-miR-486-5p	8.21E-10	EHHADH
Valine, leucine and isoleucine biosynthesis	hsa-let-7 f-5p	0.00010774	BCAT1
	hsa-let-7i-5p	0.00017172	BCAT1
Signaling pathways regulating pluripotency of stem cells	hsa-let-7 f-5p	2.97E-05	DVL3, HOXB1, HAND1, SMARCAD1, IGF1R, SKIL, ACVR1C
	hsa-let-7i-5p	0.00146009	HOXB1, HAND1, SMARCAD1, IGF1R, SKIL, ACVR1C
ECM-receptor interaction	hsa-miR-181a-5p	0.01606579	SPP1
	hsa-miR-486-5p	0.00227486	COL6A6
	hsa-let-7i-5p	0.00146009	COL1A2
Steroid hormone biosynthesis	hsa-miR-30a-5p	5.55E-07	UGT2A3
Metabolic pathways	hsa-miR-486-5p	2.74E-05	PGAP1, PYCR2, CRLS1, ST6GALNAC6, HSD17B2, EHHADH
Valine, leucine and isoleucine degradation	hsa-miR-486-5p	0.00096197	EHHADH
GABAergic synapse	hsa-miR-486-5p	0.00129123	SLC38A1, GABRB3
	hsa-miR-27a-3p	0.00765625	PRKX, GABRP, SLC6A1

### Targeted genes by multiple differentially abundant miRNAs

**Figure 7.**
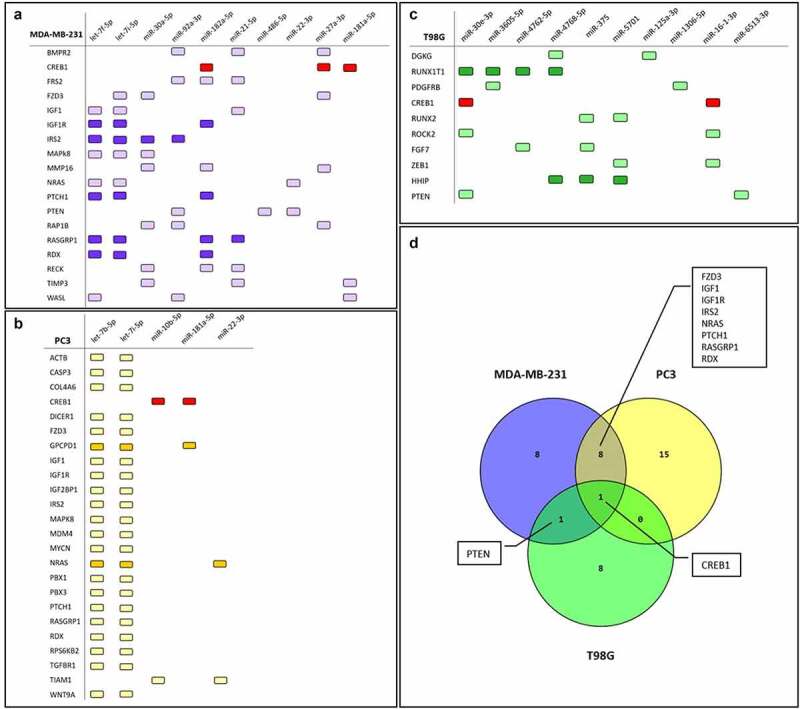
**Targeted genes by differentially abundant miRNAs**. List of genes targeted by multiple differentially abundant miRNAs in MDA-MB-231 (a), PC3(b) and T98G (c) cells subjected to the IS. In darker purple squares (A), the genes targeted by 4 miRNAs or the same 3 miRNAs. In darker yellow squares (B), the genes targeted by 3 miRNAs. In darker green squares (C), the genes targeted by 3 or 4 miRNAs. In red squares CREB gene, in common with all the cancer cell lines. In D) the Venn diagram shows the targeted genes in common among the 3 cancer cell lines

From the total list of target genes, we have identified several genes targeted by multiple differentially abundant miRNAs in the 3 cell lines subjected to the IS. In Figure 7a, we report the list of 18 genes targeted by 3 or more miRNAs in the MDA-MB-231 breast cancer cell line. A combination of 4 different miRNAs target the IRS2 and RASRGP1 genes in the MDA-MB-231 breast cancer cell line (Figure 7a, darker purple squares). IGF1R, PTCH1, RASGRP1 and RDX are targeted by a combination of the same 3 miRNAs (Figure 7a, darker purple squares), while all the rest of the 16 genes were targeted, each, by different combinations of 3 miRNAs.

Figure 7b and c shows the list of 24 and 10 genes targeted by at least 2 or more miRNAs found differentially abundant in the PC3 prostate and T98G glioblastoma cancer cell lines, respectively. In PC3 prostate cells, most of the identified genes are targeted by the combination of let-7b-5p and let-7i-5p as shown in Figure 7b. The GPCPD1 and NRAS genes were hit by a combination of 3 miRNAs (Figure 7b, darker yellow squares). In T98G glioblastoma cell line we found a combination of 4 miRNAs (miR-30e-3p, miR-3605-5p, miR-4762-5p, miR-4768-5p) which are targeting the same gene, RUNX1T1, and a combination of 3 miRNAs (miR-4768-5p, miR-375, miR-5701) targeting the HHIP gene (Figure 7c, darker green squares). Furthermore, the combination of miR-16-1-3p and miR-30e-3p is targeting, simultaneously, the CREB and ROCK2 gene (Figure 7c). Overall, MDA-MB-231 and PC3 cancer cell lines share 9 targeted genes such as CREB1, FZD3, IGF1, IGF1R, IRS2, MAPK8, NRAS, PTCH1, RASGRP1 and RDX (Figure 7d). T98G has a complete different set of targeted genes except for the PTEN gene that shares with the MDA-MB-231 cells and CREB1 gene which is shared by all 3 cell lines (Figure 7a, b and C, red squares).

### Gene validation with website tools

**Figure 8.**
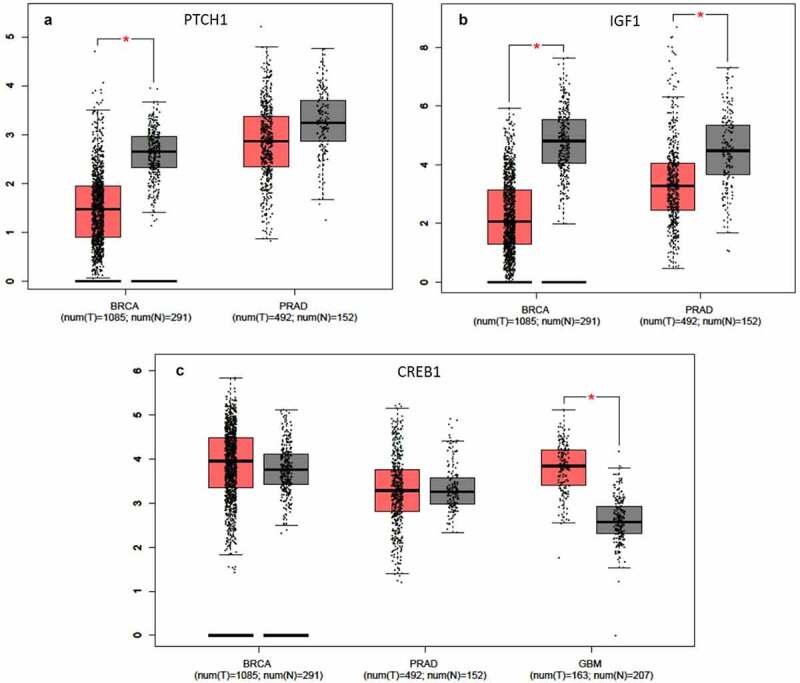
**Gene validation with GEPIA tool**. Gene expression levels box plots in tumor (red) and healthy (gray) patients are shown. (a) PTCH1 expression level in breast cancer (BRCA) and prostate adenocarcinoma (PRAD) cohorts. (b) IGF1 expression level in breast cancer (BRCA) and prostate adenocarcinoma (PRAD) cohorts. (c) CREB1 expression level in breast cancer (BRCA), prostate adenocarcinoma (PRAD) and glioblastoma (GBM) cohorts. The GEPIA dataset included 1085 breast cancers and 291 normal breast tissues; 492 prostate cancers versus 152 normal prostate tissues; 163 glioblastoma samples and 207 normal brain tissues

We further investigated the genes targeted by multiple differentially abundant miRNAs by the use of the website tool GEPIA. Initially, we loaded on GEPIA the 9 targeted genes in common between MDA-MB-231 and PC3 cancer cell lines (Figure 7d). We focused our attention on 2 genes, PTCH1 and IGF1, and we compared the expression of both genes in 1085 breast cancers versus 291 normal breast tissues and 492 prostate cancers versus 152 normal prostate tissues (Figures 8a and 8b) Both genes resulted significantly downregulated in the tumor group compared to the normal one except for the PTCH1 gene in prostate cancer (Figure 8a).

Furthermore, we decided to investigate the expression of CREB1, which, among the three cell lines, is the only gene in common targeted by the exosome-derived miRNAs (Figure 7d). We observed a slight increase of expression in the breast and prostate tumor populations which did not reach statistical significance as shown in Figure 8c. The same gene, however, was significantly up-regulated when we interrogated 163 glioblastoma samples vs 207 normal brain tissues (GEPIA tool) (Figure 8c). It is important to pinpoint that in the IS stimulated breast and prostate cancer cell lines the miRNAs targeting the CREB gene are all more abundant than in control cells. On the contrary, in the glioblastoma tumor group, where the upregulation of CREB reaches significance, the 2 miRNA targeting the gene are less abundant respecting the common downregulating action of miRNAs.

**Figure 9.**
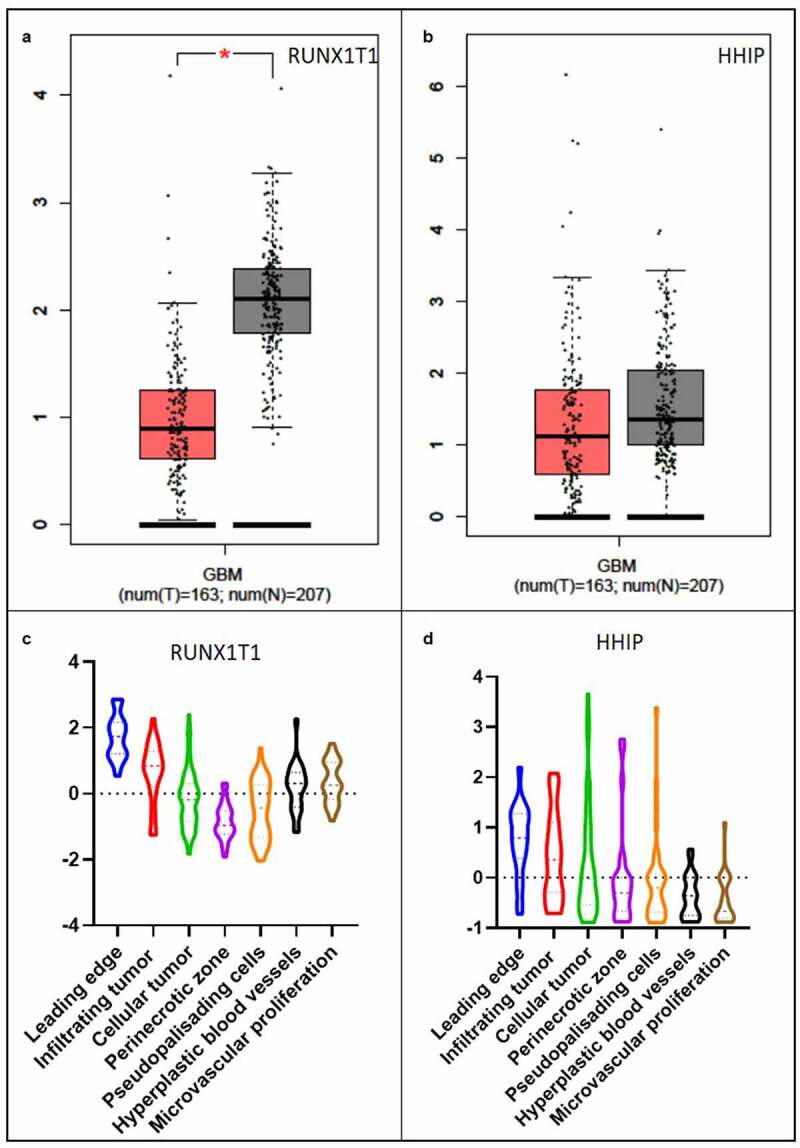
**Gene validation with GEPIA and Ivy Glioblastoma Atlas Project tool in Glioblastoma**. RUNX1T1 (a) and HHIP (b) expression level in glioblastoma (GBM) patients analyzed with GEPIA tool. Tumor samples in red and healthy samples in gray. Violin plot of RUNX1T1 (c) and HHIP (d) expression level in different tumoral portions analyzed with Ivy Glioblastoma Atlas Project tool. The Ivy database consists in 42 Infiltrating Tumor sampled by reference histology, 111 Cellular Tumor portions sampled by low expression of gene TNFAIP3, 66 Pseudopalisading cells around necrosis sampled by high expression of gene TNFAIP3, 22 Hyperplastic blood vessels portions in cellular tumor sampled by high expression of gene TGFBR2 and 28 Microvascular proliferation sampled by high expression of gene TGFBR2

Finally, we focused our attention on RUNX1T1 and HHIP gene, the genes more affected by the exosome-derived miRNAs in glioblastoma cell lines subjected to invasion.

(Figure 7c). GEPIA analysis showed a significant lower expression of RUNX1T1 and HHIP in the tumor samples (n = 163) compared to the normal tissues (n = 207) (Figures 9a and 9b). However, while the downregulation of the 2 genes seems to be specific of GBM, we used the Ivy Glioblastoma Atlas Project to highlight that in the tumoral microenvironment RUNX1T1 and HHIP vary their expression according to tumoral location. In Ivy database a differential gene expression is shown on the basis of the tumoral portion, in particular taking into consideration the cellular tumor (tumor core), the microvascular proliferation, the pseudopalisading cells around necrosis and the leading edge portions. In Figures 9c and 9d it is shown the differential expressions of the RUNX1T1 and HHIP genes in different tumor portions, revealing a higher expression of both genes in the most infiltrating portions.

### MiRNA pathway analysis

#### MDA-MB-231 cancer cell line

**Figure 10.**
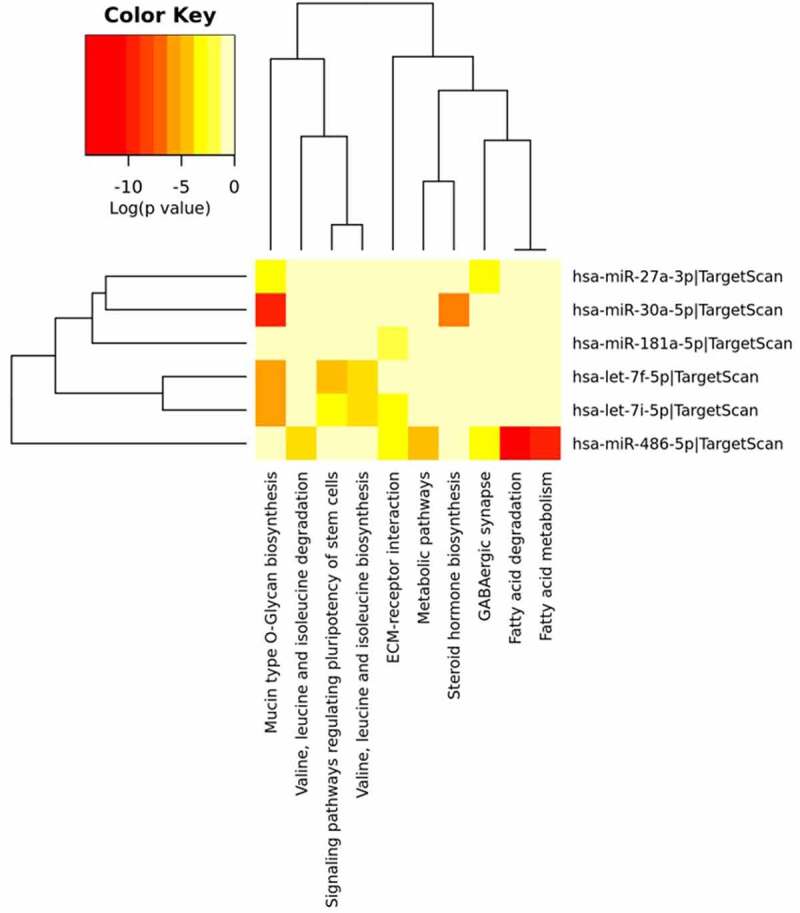
**MDA-MB-231 miRNA Pathway Analysis**. Heatmap done with mirPath v3 tool showing the pathways identified by the target genes of the differentially abundant exosomes-derived miRNAs in MDA-MB-231 cells. The first top 10 differentially abundant miRNAs were included in the software, setting the threshold for miTG score to 0.9 and doing the research among the genes related to KEGG pathways with description that matches ‘cancer invasion’

By means of mirPath v3 tool, we identified several significant molecular pathways that involve the target genes of the differentially abundant miRNAs. With the top 10 most significant miRNAs, in MDA-MB-231, we identified 10 molecular pathways, all significantly affected by 6 miRNAs as shown in Figure 10. The most significant affected molecular pathway is that of *Mucin type O-glycan biosynthesis* in which three genes, targeted by 4 different miRNAs, are involved and belong to the GALNT family proteins (Table 4). The second two most significant pathways identified are those of the *Fatty acid Metabolism*, which involve the same EHHADH gene for both pathways targeted by miR-486-5p (Figure 10). The EHHADH gene is involved, with much less significance, also in the *Valine, leucine and isoleucine degradation* pathway targeted by the same miRNA. The pathway *Valine, leucine and isoleucine biosynthesis*, and *Signaling pathways regulating pluripotency of stem cells* are targeted by the same 2 miRNAs belonging to the same family (let-7) but several different genes are involved as reported in Table 4 and Figure 10. MiR-486-5p targets other two pathways such as the *Metabolic* and *Gabaergic synapse pathways* involving, however, different genes. The *Steroid hormone biosynthesis* is targeted by a one miRNA (miR-27a-3p) and involves the UGT2A3 gene.

#### PC3 cancer cell line

**Figure 11.**
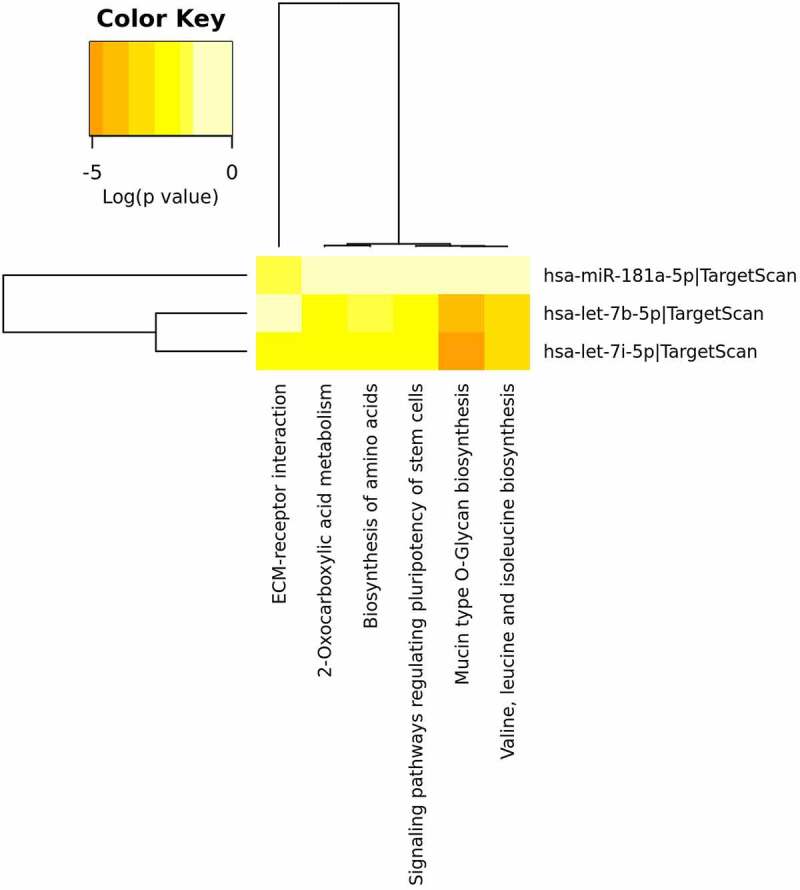
**PC3 miRNA Pathway Analysis**. Heatmap done with mirPath v3 tool showing the pathways identified by the target genes of the differentially abundant exosomes-derived miRNAs in PC3 cells. The 6 differentially abundant miRNAs were included in the software, setting the threshold for miTG score to 0.9 and doing the research among the genes related to KEGG pathways with description that matches ‘cancer invasion’

In PC3 cell line, 6 molecular pathways resulted significantly targeted by 3 different differentially abundant miRNAs such as let-7b-5p, let-7i-5p and miR-181a-5p. Four out of 6 are hit by the same 2 miRNAs (Figure 11, Table 5). The *Mucin type O-glycan biosynthesis* pathway was the most significant targeted pathway with the involvement of the GALNT1 gene (Table 5). As shown in Figure 11, 3 molecular pathways such as *Valine, leucine and isoleucine biosynthesis, 2-Oxocarboxylic acid metabolism* and *Biosynthesis of amino acids* were involved in the metabolism and biosynthesis of amino acids involving the BCAT1 gene as well as described in the MDA-MB-231 cells. Several genes resulted to be involved in the targeting of the *Signaling pathways regulating pluripotency of stem cells* such as: HOXB1, HAND1, SMARCAD1, IGFR1, SKIL and ACVR1 (Table 5).

**Table 5.** **Targeted pathways in PC3 prostate cancer cell line**. List of significantly targeted molecular pathways with genes involved

KEGG Pathway	miRNA	p Value	Genes
Mucin type O-Glycan biosynthesis	hsa-let-7b-5p	1.20E-05	GALNT1
	hsa-let-7i-5p	8.19E-06	GALNT1
Valine, leucine and isoleucine biosynthesis	hsa-let-7b-5p	2.04E-04	BCAT1
	hsa-let-7i-5p	0.000171723	BCAT1
Signaling pathways regulating pluripotency of stem cells	hsa-let-7b-5p	0.00258016	HOXB1, HAND1, SMARCAD1, IGF1R, SKIL, ACVR1C
	hsa-let-7i-5p	1.46E-03	HOXB1, HAND1, SMARCAD1, IGF1R, SKIL, ACVR1C
ECM-receptor interaction	hsa-let-7i-5p	1.46E-03	COL1A2
	hsa-miR-181a-5p	1.61E-02	SPP1
2-Oxocarboxylic acid metabolism	hsa-let-7b-5p	7.28E-12	BCAT1
	hsa-let-7i-5p	5.12E-03	BCAT1
Biosynthesis of amino acids	hsa-let-7b-5p	0.011430774	ARG2, BCAT1
	hsa-let-7i-5p	0.007808806	ARG2, BCAT1

#### T98G cancer cell line

**Figure 12.**
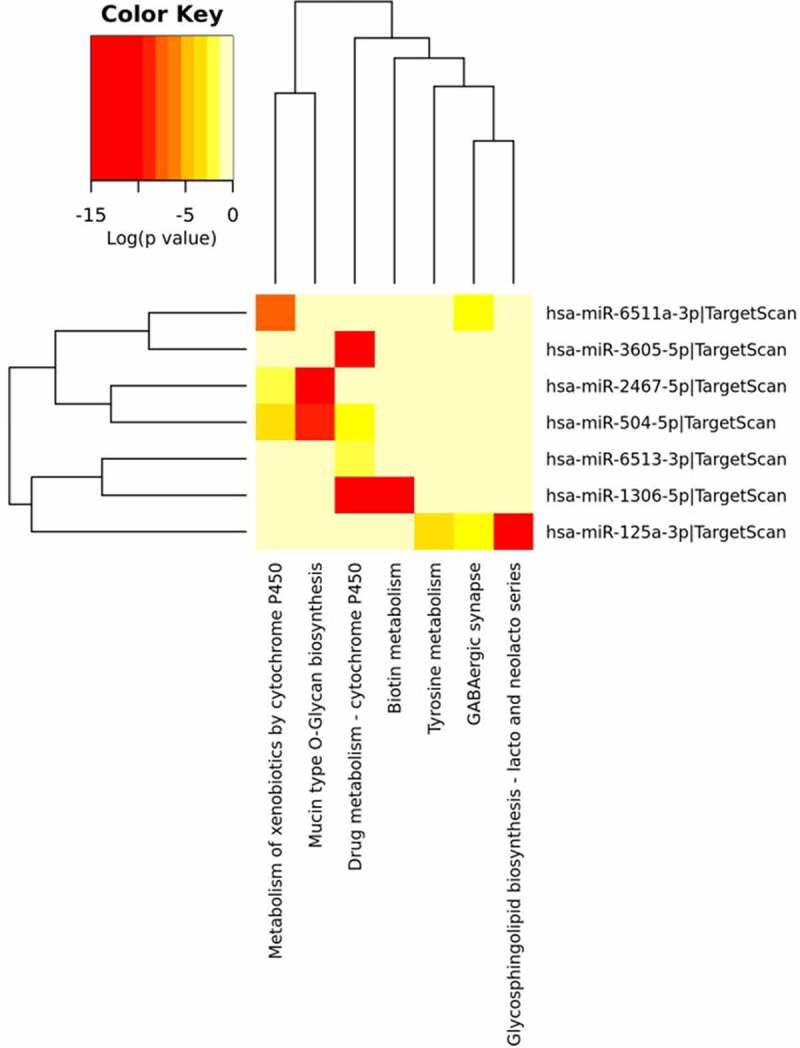
**T98G miRNA Pathway Analysis**. Heatmap done with mirPath v3 tool showing the pathways identified by the target genes of the differentially abundant exosomes-derived miRNAs in T98G cells. The first top 10 differentially abundant miRNAs were included in the software, setting the threshold for miTG score to 0.9 and doing the research among the genes related to KEGG pathways with description that matches ‘cancer invasion’

Seven molecular pathways resulted significantly targeted by differentially abundant miRNAs in T98G glioblastoma cell line (Figure 12). As well as in the breast and prostate cancer cell lines, here the most significant pathway is represented by the *Mucin type O-glycan biosynthesis* one. However, in this case, it is targeted by different miRNAs such as miR-2467-5p and miR-504-5p involving, in addition to the GALNT genes family, the POC1B gene (Figure 12, Table 6). Several miRNAs are significantly targeting, in different combinations, 2 pathways involving the metabolism of drugs and xenobiotics regulated mostly by cytochrome P450 genes family (CYP3A4, CYP3A4, CYP2B6) (Table 6). MiR-125a-3p significantly regulates three different molecular pathways such as the *Tyrosine metabolism, Glycosphingolipid biosynthesis-lacto and neolacto series* and *GABAergic synapse* pathways targeting in each a different gene, ALDH3B1, FUT6, and GABR gene family respectively (Figure 12, Table 6). As shown in Figure 12 and Table 6, the *Biotin metabolism* pathway is significantly controlled by miR-1306-5p, which targets the HLCS gene.

**Table 6.** **Targeted pathways in T98G glioblastoma cancer cell line**. List of significantly targeted molecular pathways with genes involved

KEGG Pathway	miRNA	p Value	Genes
Mucin type O-Glycan biosynthesis	hsa-miR-2467-5p	2.22E-18	POC1B-GALNT4, GALNT4, GALNT8
	hsa-miR-504-5p	9.65E-10	GALNT14, GALNT10
Drug metabolism – cytochrome P450	hsa-miR-1306-5p	1.12E-18	ADH4, CYP3A4
	hsa-miR-6513-3p	0.018682565	ADH7
	hsa-miR-3605-5p	2.36E-25	CYP3A4
	hsa-miR-504-5p	0.005229361	GSTO2, ADH1B, CYP2B6
Metabolism of xenobiotics by cytochrome P450	hsa-miR-2467-5p	0.010550422	GSTM4, ALDH3B2
	hsa-miR-6511a-3p	5.42E-08	MGST2
	hsa-miR-504-5p	0.000128886	GST02, ADH1B, CYP2B6
Glycosphingolipid biosynthesis – lacto and neolacto series	hsa-miR-125a-3p	3.95E-14	FUT6
Biotin metabolism	hsa-miR-1306-5p	7.28E-12	HLCS
Tyrosine metabolism	hsa-miR-125a-3p	0.000171445	ALDH3B1
GABAergic synapse	hsa-miR-125a-3p	1.42E-03	GABRP
	hsa-miR-6511a-3p	0.008508015	GABRG2

## Discussion

It is currently well known how a class of small non-coding RNAs, miRNAs, is conserved among the species, expressed in different tissues and cell types and involved in almost every biological process [6,15,16]. Consequently, it is not surprising that miRNAs deregulation is a hallmark of several pathological conditions, including cancer [17]. MiRNAs play different roles in cancer and can have distinct functions in regulating migration, invasion, metastasis, and proliferation [18].

One of their key function is also to be able to coordinate intercellular communication with neighboring and distant cells [19]. Probably, cancer cells take advantage of this communication to reprogram target cells in cancer-associated processes such as angiogenesis, proliferation, migration, invasion and metastasis formation [14].

Various studies indicate that miRNAs can behave both as oncogenic miRNAs (by targeting tumor suppressor genes) and tumor suppressive miRNAs (by targeting oncogene) [20]. The discrepancy observed studying the literature about miRNAs functions, is evident, and, indeed, sometimes, opposite functions were observed for one miRNA even in the same tissue, and this is because there may be several mRNAs targets in specific cell types and in specific cell conditions. MiRNAs, through their action, underline a synergy between oncogenes and tumor suppressor genes (TSG) that creates a network that modulates different processes during tumorigenesis and tumor progression [20].

In this work, for the first time, we identified exosome-derived miRNAs involved in the regulation of the invasion process, potentially acting as extra and intracellular messengers. In particular, we identified the miRNAs contained in the exosomes released in the cellular medium of cells subjected to an invasion stimulus, and potentially involved in the inter-cellular cross-talks (Figure 1). Furthermore, we demonstrated that the medium containing exosomes released by cells subjected to the invasion stimulus caused a significant statistical increase in wound closure in cells during the invasion process compared to the medium derived from cells in confluency (Figures 2 and 3). In different types of cancer cell lines, we discovered inside the cells’ exosomes several miRNAs differentially abundant in a significant way in response to the application of the invasion stimulus compared to miRNAs contained in exosomes released from non-stimulated confluent cells.

Several specific exosome-derived miRNAs were identified as statistically differentially abundant in each cancer cell line tested. Overall, the total collection of differentially abundant miRNAs showed a similar trend in the three cell lines as reported in the hierarchical clustering of Figure 4. The clustering analysis of the miRNAs expression values showed a perfect grouping of the various triplicates in the correct belonging class as shown in Figure 4. However, the differentially abundant miRNAs that reached the statistical significance were different for each cell line (Figure 5a, b and c) except for the MDA-MB-231 and the PC3 cell lines that shared 3 miRNAs in common (Figure 5d and Figure 6). Finding identical miRNAs in MDA-MB-231 and in PC3 highlights a similarity between prostate and breast cancer that has already been emphasized in the past [21]. Although these tumors originate in organs that are different from an anatomical point of view, one of the elements that connect them is the involvement of sex steroid hormones in the tumor development. Therefore, it is possible that being similar, these tumors may have similar characteristics also in the way of communicating. In support of this, it has been seen that in some cases prostate and breast cancer can co-occur in families, in fact there are studies showing a statistically significant association between familial breast cancer and prostate cancer risk [22,23]. Interestingly, the more abundant miRNAs in common by PC3 and MDA-MB-231 cell lines, which were not observed in the T98G, that is miR-181a-5p, miR-22-3p and let-7i-5p, showed some conflicting results in the literature regarding their involvement in invasion. MiR-181a-5p has a dual regulatory role, acting both as an oncogene and as TSG in breast cancer [24]. However, Shen *et al*. in 2018 show how miR-181a-5p when is down-regulated inhibits proliferation and cell cycle in prostate cancer [25], in accordance with our results which report higher level during invasion (Figure 7b). In addition, miR-22-3p, already known largely in the literature for its involvement in cancer, behaves both as TSG and as oncogene to cause or prevent cancer formation and malignant transformation. However, in accordance with our results the up-regulation of miR-22-3p in the cells promotes prostate cancer formation [26] and let-7i-5p was found up-regulated in the liquid biopsy of breast cancer compare to controls, representing a potential biomarker, prognostic indicator, and therapy target for precision medicine in cancer [27]. It is well known that there are 12 different let-7 family members (let-7a-1, 7a-2, 7a-3, 7b, 7 c, 7d, 7e, 7 f-1, 7 f-2, 7 g, 7i, and miR-98) expressed in human [28]. In addition, let-7 family’s members can act like TSG or like oncogenes. Ma *et al*. [29] demonstrated that let-7e affects migration and invasion of esophageal squamous cell carcinoma cells. Moreover, let-7 f and let-7e have been shown to be up-regulated in tongue squamous carcinoma [30]. In addition, in gastric cancer the oncogenic role for let-7 family was observed: let-7 derived from exosomes was over-expressed in highly metastatic gastric tumors [31]. Let-7i [32–34] also seems to act as TSG, in gastric cancer, bladder cancer and glioblastoma. Furthermore, it is very interesting to pinpoint that in the MDA-MB-231 cell line most of the differentially abundant miRNAs were present with high levels in exosomes except for miR-486-5p which, in accordance with our findings, was recently reported acting as a tumor suppressor playing a vital role in cell apoptosis, cell growth and migration during breast cancer development [35]. Several of the more abundant miRNAs that we identified in the MDA-MB-231 cell line (Figure 5a) were recently confirmed to be oncomiRNAs by several other groups [36–44], however miR-151a-3p and miR-16-5p show conflicting results in the literature [45–47]. In this work, miR-26a-5p appears, for the first time, to be significantly more abundant in the exosomes of breast cancer cells, in contrast with a recent study where this miRNA results down-regulated in both breast cancer tissues and cell lines and shows a role in inhibiting the growth of breast cancer cells [48]. This discrepancy could be due to the fact that we are investigating a different cellular phenomenon such as invasion vs growth. Actually, Liu *et al*. in 2012 [49], describes an opposite role of miR-26a in tumor invasion or metastasis where its expression was higher in lymph node metastasis tumor tissues than in primary tumor tissues. Here, the ectopic over-expression of miR-26a dramatically enhanced lung cancer cell migration and invasion abilities supporting our findings in breast cancer cells [49].

In our study, we observed in T98G glioblastoma cells, subjected to the IS, a pattern of differentially abundant exosome-derived miRNAs completely different from the breast and prostate cancer cells (Figure 5c). Both oncogenic and tumor suppressive miRNAs were found to affect cell invasion, in particular in our findings we observed several miRNAs whose trend is confirmed by the available literature such as miR-375, that inhibits proliferation and invasion in glioblastoma [50] and also inhibits the migration in gastric cancer. Besides, miR-5701 has an anti-tumor role in cervical cancer [51] and miR-16-1 inhibits migration and proliferation in glioma cells [52]. Still in accordance with our results, miR-125a behaves as a TSG, in glioma cells, inhibiting cell invasion [53]. With regard to miR-30-e, a study shows that the expression of this miRNA is down-regulated when compared with the larger necrotic volume group identified by MRI in GBM patients [54]. In addition, miR-628-3p was less abundant in glioblastoma cells after the IS, as reported in the literature where it is found down-regulated in the blood of glioblastoma patients and suggested as a potential diagnostic biomarker for poor prognosis [55]. We found in the literature conflicting results related to miR-504-5p, miR-4762-5p, miR-4768-5p, and miR-3605-5p [56–58], while miR-6511a, miR-6513-3p, miR-1306-5p and miR-2467-5p have not been described before in glioblastoma cells.

It is crucial to possess a method to identify precisely the miRNA targets, prior to applying any experimental approaches that allow a better functional characterization of miRNAs in biological processes and can thus predict their effects. We used a rapid computational prediction tool, mirPAth v3, to identify putative miRNA targets. By analyzing the statistically significant differentially abundant miRNAs in each cancer cell line, we were able, in a first phase, to identify several targeted genes. It is experimentally confirmed that multiple miRNAs can target the same genes, suggesting that it is the combination of all these activities that should lead researchers to focus on networks more than on individual connections between miRNAs and strongly predicted targets [59]. In Figure 7 we show the most significant targeted genes controlled by the multiple differentially abundant miRNAs identified after the IS was administered to MDA-MB-231, PC3 and T98G cells. We decided to focus on the genes that were controlled simultaneously by multiple miRNAs that we found significantly differentially abundant. This is because we consider that they can represent the main molecular pathways through which the cell carries out its action, and therefore more finely regulated and maneuvered. It is worth noticing that the MAD-MB231 and PC3 cells share not only few miRNAs but also several genes, which ended up being targeted also by different miRNAs, as shown in Figure 7d. This strongly suggests the potential leading role of these genes in the invasion process in both breast and prostate cancer cell systems. Concerning miRNA-FZD3, one report has been published about breast cancer and knockdown of FZD3 decreased MDA-MB-231 cell invasion [60]. IGF1 is a potent mitogen of relevance in the mammary gland. IGF1 binding to the cognate receptor, IGF1R, triggers a signaling cascade that causes proliferative and anti-apoptotic events. A growing body of evidence leads to the important role of the IGF1 system in breast cancer development, progression and metastasis. IGF1 is a point of convergence for major signaling pathways implicated in breast cancer growth [61]. Insulin receptor substrate 2 (IRS2), that we found targeted simultaneously by 4 miRNAs, is a candidate driver oncogene frequently amplified in cancer with the ability to modulate and coordinate multiple signaling cascades, transmitting upstream signals to intracellular pathways, including the PI3K/AKT/mTOR and MAP kinase pathways [62]. *In vivo* and in vitro experimental models have highlighted the role of increased insulin and IGF signaling in enhancing tumorigenesis [63]. The RASRGP1 gene is the second gene that in MDA-MB-231 cells resulted to be targeted by 4 different miRNAs, 3 of which target also the IGF-1 R and PTCH1 genes. RasGRP proteins are activators of Ras and other related small GTPases by the virtue of functioning as guanine nucleotide exchange factors and only in recent studies it is demonstrated that RasGRPs also plays an important role in tumorigenesis [64]. PTCH1 and RDX are other 2 genes shared by our breast and prostate cancer cells, both are targeted by multiple miRNAs. It is well known in literature the role of PTCH1 as a TSG in several tumors such as medulloblastoma and basal cell carcinoma [65] . The RDX gene codes for one of the four proteins involved in the actin cytoskeleton pathway, and has been identified as a let-7 family direct target. Blocking the expression of RDX significantly inhibits breast cancer cell migration regulated by let-7b repression [66]. Two genes resulted targeted by 3.

miRNAs in prostate cancer, the GPCPD1 and NRAS gene, and the same combination of let-7b-5p and let-7i-5p were involved. Altered choline metabolism is a hallmark of cancer. More specifically, a decreased glycerophosphocholine (GPC) to phosphocholine (PC) ratio was reported in breast, ovarian, and prostate cancers [67], suggesting a tumor suppressor role for this gene in cancer. The NRAS gene is a well know oncogene in cancer that encodes for a GTP binding intracellular protein that interacts with the EGFR receptor and in 2018 has proposed as a possible biomarker for advanced prostate cancer [68].

In order to understand the role of the exosomes-derived miRNAs of the cancer cells, subjected to IS, in affecting target genes, we analyzed their gene expression using specific cancer databases (GEPIA). For MDA-MB-231 and PC3 we focused on PTCH1 and IGF1 gene, described before. According to our results, the exosome-derived miRNAs affecting PTCH1 and IGF1genes (let-7 f-5p, let-7i-5p, miR-182a-5p and miR21-5p for MDA-MB-231 and let-7b-5p and let-7i-5p for PC3) were all more abundant in the cells subjected to IS than in the control, suggesting to act as gene silencers during invasive conditions. In agreement with this, the analyses performed using the GEPIA tool confirm that in tumor patients the expression of PTCH1 and IGF1 genes is lower than in normal patients, suggesting therefore a downregulation in a more aggressive cell state (Figures 8a and 8b).

From our findings, the pattern of genes, as well as miRNAs, involved in cell invasion in glioblastoma cells appears to rotate around different hinges from those seen in breast and prostate cells. The most targeted genes are represented by the RUNX1T1 and the HHIP genes. RUNX1T1 has been extensively involved in multiple cancers, it encodes a member of the myeloid translocation gene family which interact with DNA-bound transcription factors and recruit a range of corepressors to facilitate transcriptional repression having, therefore, a suppressive function in cancer [69]. The combination of differentially abundant miRNAs, controlling the RUNX1T1 gene, included miR-4762-5p more abundant in the IS group that might in this case act as a tumor suppressor miRNA. Similarly, the HHIP gene, that encodes for a hedgehog-interacting protein has a tumor suppressive function in glioblastoma as Chang *et al*. reported in 2015 [70]. In support of these literature data, the analyses made with GEPIA showed lower expression levels of both genes in tumor patients (Figures 9a and 9b) in agreement with the more abundant state of miR4762-5p in the IS stimulated glioblastoma cells (Figure 5c). However, our data revealed also several miRNAs, targeting RUNX1T1 and HHIP (miR-30-e-3p, miR-3605-5p and miR-4768-5p for RUNX1T1 and miR-4768-5p, miR-375 and miR-5701 for HHIP gene) which were significantly less abundant in the exosomes of the T98G IS cells (Figure 5c) suggesting an opposite role consisting in promoting instead the overexpression of the genes. These conflicting results were solved with the results obtained by analyzing the expression of RUNX1T1 and HHIP in different tumor locations within the glioblastoma microenvironment through the use of the Ivy glioblastoma Atlas Project. We observed that despite glioblastoma is affected by a general downregulation of RUNX1T1 and HHIP compared to the normal brain tissue, in the cancer context not all portions of the tumor have a downregulation of these genes but in the more invasive portions the expression of RUNX1T1 and HHIP is higher compared to the other regions. This result recalls the crucial importance of tumor heterogeneity in the study of cancer (Figures 9c and 9d).

The CREB1 is the only gene in common among the three cancer cell lines tested after the administration of the IS. The presence of the same target gene in the three cell lines subjected to invasion suggests a key role for the CREB1 gene in the invasion process regardless of cell type. It has been described several times, in fact, how this transcription factor is involved in various cancer, raising its level in the advanced stages of the disease, being predictive of relapse and correlated with poor outcomes [71–73]. Through the analyses performed with GEPIA we did not observe a significant differential expression of the CREB1 gene in both breast and prostate patients (Figure 8c). However, for GBM patients, a significantly higher expression was detected in the tumor patients (Figure 8c) in accordance with our results, as miR-30e-3p and miR-16-1-3p affecting CREB1 were less abundant in IS T98G cells.

Utilizing the same computational method, we further statistically investigated which were the molecular pathways mostly controlled by the targeted genes and the differentially abundant miRNAs identified in the exosomes released by the IS-stimulated cells. All three cancer cell lines were characterized by the involvement of the *Mucin type O-glycan biosynthesis* pathway which resulted the mostly significantly targeted (Tables 4, Tables 5 and Tables 6). O-glycosylation is the most common modification for the mucins and the alterations in mucin glycans are observed in a lot of tumors [74]. In cancer, it is possible to observe truncated structures or aberrant extension of glycan chains [74,75]. Atypical glycan structures formed on glycoproteins are involved in cancer progression. The role of these aberrant glycans is well documented in cancer cell adhesion, motility, invasion, and altering the interaction of cancer cells with other cells, such as lymphocytes and endothelial cells [76]. Glycosylation is a key process impacting on many aspects of cellular interactions. It was recently reported that a miRNA cluster controls glycosylation by directly targeting N-acetyl galactosamine transferases (GALNTs), resulting in increased tumor invasion and immunosuppression in melanoma [77]. In our context, the miRNA clusters identified could behave exactly like in melanoma cells targeting the GALNT genes family as shown in Tables 4, Tables 5 and Tables 6. Both for MDA-MB-231 and PC3, ECM-receptor interaction pathway was observed: in fact, as it is well known, the extracellular matrix (ECM) represents the fundamental support for cells and it is responsible for cell–cell communication. The interactions between cells and the ECM are regulated by some macromolecules like the integrins and other cell-surface-associated components that control processes such as adhesion, proliferation and migration. In cancer, the ECM is the major component of the cancer microenvironment and when the tumor cells begin to proliferate, the ECM undergoes architectural changes that allow cancer progression [78]. Among the three cancer cell lines, as shown in Figures 10, 11, and 12 it is clear that the metabolism of amino acids and fatty acids is well represented. An abundant supply of amino acids and fatty acids is important for cancers to sustain their proliferative drive. Amino acids, alongside their direct role as substrates for protein synthesis, can have roles in energy generation, driving the synthesis of nucleosides and maintenance of cellular redox homeostasis, while fatty acids are essential mediators of cancer progression and metastasis, through remodeling of the tumor microenvironment [79,80].

In conclusion, cancer cells are able to release exosomes and mediate communication among each other and with other cell types. In particular, their miRNAs cargo can certainly play a key role in supporting the tumor invasion. We can highlight that in the breast and prostate cancer cell lines, tested in this work, the invasion seems mediated by the same messengers, since we found some significative miRNAs and targeted genes in common. On the other hand, the glioblastoma cell line showed a certain uniqueness of molecular mediators. Here we report for the first time the variable gene expression behavior of RUNX1T1 and HHIP related to tumor location within a glioblastoma context.

The process of regulation of gene expression driven by the action of miRNAs is very complex and, on the basis of part of our results, we confirm that the higher abundance of some miRNAs is associated with a down-regulation of the target gene, as more commonly described. However, concerning certain targeted genes, it seems necessary to also suggest a direct promotion, by the miRNA, of an over-expression rather than a down-regulation of the target genes, in accordance with the gene function extensively described in the literature.

Our work has some limitations. First of all, we used cancer cell lines in 2D to perform the experiments and surely this is different than using tissues with a real microenvironment that surrounds the cells, but our goal was to study the communication between cells as a starting point. Indeed, as a result of our observations, the study of communication between cancer cells in their tumor microenvironment could be an excellent future perspective. In addition, we decided to use only one cell line type per tumor, albeit in triplicate, instead of three different cell types per tumor.

In this work, the study of direct communication between cells of the same type has been deepened, pruning from the interference of other cellular components. Although interactions within the multi-cellular microenvironment are crucial in the invasion process, here we have moved on to the essence of communication among cells, of the same type, engaged in a very specific activity. In such a way, we can have precise information on what are the messages that the cells send each other to carry out their activity. This can provide insights for reconstructing backward an even more complex process, such as the tumor invasion of the surrounding environment in the presence of other cellular components. Therefore, understanding the language codes among cells engaged in invasion can lead to the development of therapies that can block or interfere cellular communication, slowing or eventually stopping their task.

## Supplementary Material

Supplemental Material
