# Supplementary material for: Analysis of exosome-derived microRNAs reveals insights of intercellular communication during invasion of breast, prostate and glioblastoma cancer cells
Source: Cell Adh Migr. 2021 Jun 22;15(1):180–201. doi: 10.1080/19336918.2021.1935407 (PMC8224203; doi:10.1080/19336918.2021.1935407)
Supplement: Supplemental Material [file KCAM_A_1935407_SM6635.zip › supplementary/Supplementary File 1.docx]

;General parameters

[General]

; type of analysis (miRNA, mRNA or circRNA)

type=miRNA

; Folder for miRNA reads

read_dir=Examples/basic_examples/miRNAs/InputFiles/

; label for the analsysis

label=MiRNAEsosomi

; Folder where miARma has been instaled

miARmaPath=.

; Folder to store results

output_dir=Examples/basic_examples/miRNAs/OutputFiles/

; organism used

organism=human

; Number of process to run at the same time

threads=24

; Whether the data is from a strand-specific assay (yes, no or reverse, yes by default) for featureCounts analysis

strand=yes

stats_file=Examples/basic_examples/miRNAs/Known_miRNAs/results//miARma_stat.120866.log

logfile=Examples/basic_examples/miRNAs/Known_miRNAs/results//miARma_logfile.120866.log

[Quality]

prefix=Both

[Adapter]

; Adapter sequence to be removed in the analysis

adapter=TGGAATTCTCGGGTGCCAAGG

; Specific software to remove the adapter from the sequences

adaptersoft=CutAdapt

[Aligner]

; Aligner (Bowtie1, Bowtie2, BWA or miRDeep)

aligner=Bowtie1

; Bowtie 2 index

bowtie1index=Genomes/Indexes/bowtie1/human/bw1_homo_sapiens19

[ReadCount]

#GFF file used to calculate the number of reads in featureCounts analysis

database=Examples/basic_examples/miRNAs/data/miRBase_Annotation_20_for_hsa_mature_miRNA.gtf

;GFF attribute to be used as feature ID (default: gene_id) for featureCounts analysis

seqid=transcript_id

;Feature type (3rd column in GFF file) to be used, all features of other type are ignored (default:exon) for featureCounts analysis

featuretype=miRNA

[DEAnalysis]

; Complete path of the target file.

targetfile=Examples/basic_examples/miRNAs/data/targets.txt

; Path of the contrast file.

contrastfile=Examples/basic_examples/miRNAs/data/contrastEso.txt

#This value refers to filter processing in the reads (Should be "yes" or "no").

filter=yes

;Specific software to perform the Differential Expression Analysis (Allowed values: edger, noiseq or edger-noiseq)

desoft=edger

;Provide a file with normalized reads

cpm=yes

;Provide a file with RPKM values

rpkm=yes

[TargetPrediction]
