# Supplementary material for: Analysis of exosome-derived microRNAs reveals insights of intercellular communication during invasion of breast, prostate and glioblastoma cancer cells
Source: Cell Adh Migr. 2021 Jun 22;15(1):180–201. doi: 10.1080/19336918.2021.1935407 (PMC8224203; doi:10.1080/19336918.2021.1935407)
Supplement: Supplemental Material [file KCAM_A_1935407_SM6635.zip › supplementary/Tables_captions.docx]

Table 1S. Exosome-derived differentially abundant miRNAs data. Quality of the sequencing and reads obtained.

Table 2S. List of the genes targeted by the differentially abundant exosome-derived miRNAs for MDA-MB-231 cell line obtained with the DIANA-microT tool.

Table 3S. List of the genes targeted by the differentially abundant exosome-derived miRNAs for PC3 cell line obtained with the DIANA-microT tool.

Table 4S. List of the genes targeted by the differentially abundant exosome-derived miRNAs for T98G cell line obtained with the DIANA-microT tool.
